# Response of the Abundance of Key Soil Microbial Nitrogen-Cycling Genes to Multi-Factorial Global Changes

**DOI:** 10.1371/journal.pone.0076500

**Published:** 2013-10-04

**Authors:** Ximei Zhang, Wei Liu, Michael Schloter, Guangming Zhang, Quansheng Chen, Jianhui Huang, Linghao Li, James J. Elser, Xingguo Han

**Affiliations:** 1 State Key Laboratory of Forest and Soil Ecology, Institute of Applied Ecology, Chinese Academy of Sciences, Shenyang, China; 2 State Key Laboratory of Vegetation and Environmental Change, Institute of Botany, Chinese Academy of Sciences, Beijing, China; 3 State Key Laboratory of Systematic and Evolutionary Botany, Institute of Botany, Chinese Academy of Sciences, Beijing, China; 4 Environmental Genomics, Helmholtz Center for Environmental Health, Oberschleissheim, Germany; 5 School of Life Sciences, Arizona State University, Tempe, Arizona, United States of America; INRA Clermont-Ferrand Research Center, France

## Abstract

Multiple co-occurring environmental changes are affecting soil nitrogen cycling processes, which are mainly mediated by microbes. While it is likely that various nitrogen-cycling functional groups will respond differently to such environmental changes, very little is known about their relative responsiveness. Here we conducted four long-term experiments in a steppe ecosystem by removing plant functional groups, mowing, adding nitrogen, adding phosphorus, watering, warming, and manipulating some of their combinations. We quantified the abundance of seven nitrogen-cycling genes, including those for fixation (*nifH*), mineralization (*chiA*), nitrification (*amoA* of ammonia-oxidizing bacteria (AOB) or archaea (AOA)), and denitrification (*nirS*, *nirK* and *nosZ*). First, for each gene, we compared its sensitivities to different environmental changes and found that the abundances of various genes were sensitive to distinct and different factors. Overall, the abundances of nearly all genes were sensitive to nitrogen enrichment. In addition, the abundances of the *chiA* and *nosZ* genes were sensitive to plant functional group removal, the AOB-*amoA* gene abundance to phosphorus enrichment when nitrogen was added simultaneously, and the *nirS* and *nirK* gene abundances responded to watering. Second, for each single- or multi-factorial environmental change, we compared the sensitivities of the abundances of different genes and found that different environmental changes primarily affected different gene abundances. Overall, AOB-*amoA* gene abundance was most responsive, followed by the two denitrifying genes *nosZ* and *nirS*, while the other genes were less sensitive. These results provide, for the first time, systematic insights into how the abundance of each type of nitrogen-cycling gene and the equilibrium state of all these nitrogen-cycling gene abundances would shift under each single- or multi-factorial global change.

## Introduction

Nitrogen (N) cycling is one of the most important ecosystem functions and most soil N-cycling processes are driven by microbes [1]–[4]. For example, N fixing microbes convert N_2_ into NH_4_^+^, providing most of the N ultimately used by organisms on Earth [5], [6]. In turn, mineralizing microbes decompose organic N into NH_4_^+^, most of which will be reused by organisms [7], while nitrifying microbes oxidize NH_4_^+^ into NO_2_^-^ and then NO_3_^-^, often a preferable N form for plants [8], [9]. Denitrifying microbes reduce NO_3_^-^ into NO, N_2_O and N_2_, returning N to the atmosphere and completing the entire N-cycling process [10], [11]. As anthropogenic activities intensify, these microbe-mediated N-cycling processes are being affected by various environmental changes, often leading to undesirable consequences [12]–[15]. For example, N fertilization/deposition stimulates the production of excess NO_3_^-^ by nitrifying microbes. This excess NO_3_^-^ can be easily lost from soil by leaching, resulting in pollution of groundwater and eutrophication of lakes, estuaries, and coastal oceans [12], [14]. Therefore, the influence of various environmental changes on each type of N-cycling functional group has been widely investigated during the past several decades [16]–[19]. However, any given ecosystem can be disturbed by many kinds of environmental changes at the same time [8], [12], [20] and different environmental changes (and their concurrence) might have different influences on a given N-cycling functional group. For example, it was found that the abundance of AOB decreased under elevated CO_2_ but increased under elevated precipitation, and that the decrease under elevated CO_2_ was even more pronounced when elevated precipitation concurred with elevated CO_2_ [8]. Such studies suggest that for each type of N-cycling functional group, we should compare its sensitivity to different environmental changes. To detect or predict possible shifts of a given N-cycling process under multiple environmental changes in the future, our effort should be focused on the environmental change to which the particular microbial process of interest is more sensitive.

Although the transformations of different forms of N are driven by different microbial functional groups, equilibrium transformations of various forms of N are often achieved in pristine and mature ecosystems, leading to the maintenance of ecosystem productivity and satisfactory water quality [21], [22]. Because these microbial groups likely have different diversity, metabolic versatility and environmental tolerances [5], [8], [23], they might respond differently to a given environmental change, resulting in the shift in the equilibrium state of various N-cycling processes. Therefore, we should also investigate the sensitivities of different types of N-cycling processes in response to each single- or multi-factorial environmental change.

To systematically compare the difference in the sensitivities among different environmental changes and different N-cycling functional groups, we conducted four five-year experiments in a steppe ecosystem in Inner Mongolia of China, an area representative of much of the massive Eurasian steppe region floristically and ecologically [24], [25]. Many types of environmental changes have been influencing this steppe ecosystem simultaneously, including altered precipitation regimes, rising mean temperature, N deposition, N and P fertilization, loss of plant diversity, and shifts in grazing intensity [26]–[34]. Therefore, we mimicked these environmental changes and some of their combinations. Overall, there were four main experiments manipulating 1. plant functional groups (PFG); 2. mowing, N and phosphorus (P); 3. mowing, N and water; and 4. water and temperature. For each experiment, we assessed the response of the main components of the N cycle by quantifying the abundance of seven key N-cycling genes in the soil (*nifH*, *chiA*, AOB-*amoA*, AOA-*amoA*, *nirS*, *nirK* and *nosZ*) [10], [35]–[40]. Then, for each type of N-cycling functional group, we compared its sensitivities to different environmental changes. For each single- or multi-factorial environmental change, we compared the sensitivities of different N-cycling functional groups.

Here we used gene abundance as a proxy of the potential rate of various nitrogen-cycling processes, although the effectiveness of this index might be influenced by several factors. First, the detected gene might come from lytic/dormant microbial cells. Second, different genes might have different expression efficiency. Finally, different enzymes might have different functional efficiency. In spite of this, a recent study found that, among all chemical/physical and biological indexes (e.g., pH, water content, nitrate and ammonium content), gene abundance was the best predictor of soil nitrogen-cycling rates because this index integrated the information of recent environmental history and recent process activity [41]. In addition, measurement methods of process rates are much more labor-intensive than those of gene abundance (that was quantitative PCR); thus, we quantified gene abundance to represent various nitrogen-cycling rates.

## Materials and Methods

### Experimental design

To investigate the effects of various ecological factors on the steppe ecosystem, we conducted four long-term experiments in Inner Mongolia, China. Although some aspects of these experimental designs have already been described [20], [26], [27], [30], [31], we provide an integrated description here.

The first experiment was conducted near the Inner Mongolia Grassland Ecosystem Research Station, Chinese Academy of Sciences (116°42′E, 43°38′N). All plant species were classified into five PFGs based on their life forms [30], [42]–[45]. Among these PFGs, perennial rhizome grass, perennial bunchgrasses and perennial forbs comprised >99% of the aboveground biomass (Table S1). Therefore, we only investigated the effects of the three PFGs and their combinations. A full combinatorial design was employed with a total of eight (2^3^) treatments (Table S2) and with five replicates for each treatment. Specifically, four PFG diversity gradients were created by leaving 0, 1, 2 and 3 PFGs in 6 m ×6 m plots, with PFG diversity gradients of both 0 and 3 having only one treatment, and PFG diversity gradients of both 1 and 2 having 3 treatments. In other words, we took the treatment with all the three PFGs remained as the control, and then the treatments with 0, 1 and 2 PFGs remained would represent removing 3, 2 and 1 PFG, respectively (Table S2). PFG diversity gradients were established in July every year from 2005 to 2009 by *in situ* manual removal of the aboveground biomass of non-target plants in each plot. Stems and leaves were removed by clipping with shears or knives at the soil surface while taking great care to reduce disturbance to soil and other plants [44], [45].

The second, third, and fourth experiments were conducted in Duolun County (116°17′E, 42°02′N), near the site of the first experiment in the same steppe ecosystem [20], [26], [27], [31]. In the second experiment, the effects of mowing, N, P, and their combinations were investigated using a nested design with mowing as the primary factor and nutrient (N and P) addition as the secondary factor (Fig. S1). There were four replicates for each of the eight combinations. Within a 199 m ×265 m area, eight 60 m ×92 m primary plots were set-up with a 5-m-wide buffer zone among plots. Four primary plots were randomly assigned to the mowing treatment and the other four were controls. Aboveground biomass removal through mowing was used to mimic animal removal of grass plants. Aboveground plants were mowed on 20 August every year, leaving only 10 cm of stubble. Within each of the eight primary plots, four 28 m ×44 m secondary plots were set-up (Fig. S1), with a 1-m buffer zone between them. Each of the four secondary plots was randomly assigned to N addition, P addition, simultaneous N and P addition, and control treatments. N was added in the form of urea in 2005 and NH_4_NO_3_ in 2006–2010 at a rate of 10 g N m^-2^ y^-1^, mimicking projected N deposition rates for this region [28], [46]. P was added in the form of calcium superphosphate at a rate of 5 g PO_4_^3-^ m^-2^ y^-1^. Both N and P were added on a rainy day in the middle of July every year.

In the third experiment, the effects of mowing, N, water, and their combinations were investigated. There were four replicates for each of the eight combinations. The third experiment partially overlapped the second experiment [27]. Within each of the secondary plots treated with N addition or control, two 10 m ×15 m third-level plots were established (Fig. S1), with one third-level plot being un-watered and the other being watered during summer (July and August) in 2005–2010. Within each watered subplot, six sprinklers were evenly arranged into two rows with 5 m between rows and 5 m between adjacent sprinklers. Each sprinkler covered a circular area with a diameter of 3 m; therefore, the six sprinklers covered the 10 m ×15 m third-level plot. Fifteen millimeters of water was applied weekly; thus, in the increased precipitation treatment, about 120 mm water was added each year, which is approximately 30% of the annual mean precipitation at the study site.

In the fourth experiment, the effects of water and temperature and their combination were investigated (Fig. S1). The fourth experiment partially overlapped the third experiment [20], [26], [31]. First, we randomly selected three control (without mowing) primary plots, each of which contained a control (no added N or P) secondary plot. Within each of these three control secondary plots, there were two third-level plots, with one being watered and the other being un-watered. Within each of the six third-level plots, four 3 m ×4 m fourth-level plots were set up (Fig. S1), with two being randomly assigned to warming treatment and the other two being controls. The warmed fourth-level plots were heated continuously beginning on 28 April 2005 using 165 cm ×15 cm MSR-2420 infrared radiators (Kalglo Electronics, Bethlehem, PA, USA) suspended 2.5 m above the ground. The effects of the infrared radiators on soil temperature were shown to be spatially uniform within the warmed plots [47]. In the un-warmed control fourth-level plot, one “dummy” heater with the same shape and size as the infrared radiator was suspended 2.5 m above the plot to simulate the shading effects of the heater. From 1 August 2006 to 31 October 2007 with continuous temperature records, mean soil temperature was 1.79°C higher in the warmed plot than the control plot. Meanwhile, warming significantly reduced volumetric soil moisture at the depth of 0–10 cm by 5.0%. Overall, there were six replicates for each treatment (control, warming, watering, warming plus watering).

### Sampling and measurement of soil physicochemical indexes

For the first experiment, soil samples were taken on 22 June 2010. For the other three experiments, soil samples were taken on 22 August 2010, when aboveground plant biomass had reached its maximum height. To reduce the influence of within-plot spatial heterogeneity on the measured indexes, four soil cores (10 cm depth, 3.5 cm diameter) were taken at random locations from each plot and thoroughly mixed, part of which was used to measure soil physicochemical indexes and the rest were frozen for DNA extraction. Because the second, third, and fourth experiments were partially overlapping, for both soil physicochemical indexes and gene abundances of the overlapping plots, the mean of control plots in the third (or fourth) experiment was used to represent the corresponding indexes in the second (or third) experiment.

Soil organic carbon (C) content was analyzed by using external heating method [48]. Soil total N content was measured using an Alpkem autoanalyzer (Kjektec System 1026 Distilling Unit, Sweden) according to the Kjeldahl acid-digestion method. Soil NH_4_^+^-N and NO_3_^-^-N contents were determined on a FIAstar 5000 Analyzer (Foss Tecator, Denmark) after extraction of fresh soil with 1 mol/L KCl. Soil water content was determined as the weight loss after drying for 24 hr at 105°C. Soil pH was measured in 1∶2.5 (W/V) suspensions of soil in distilled water.

### Measurement of the abundance of various N-cycling genes

For each soil sample, we extracted DNA from 0.5 g of mixed soil according to the instructions of the Fast DNA SPIN kit for soil (Qbiogene, Carlsbad, CA, USA); however, we used 350 µL instead of 50 µL DNA elution solution to elute the DNA in the tenth step. The DNA solution was stored at −20°C.

The seven N-cycling genes were quantified using the method of real time PCR similar to previous descriptions (Table S3). For each gene, a standard curve was generated using a 10-fold serial dilution of a plasmid containing a copy of the target gene. The 25-µL PCR reaction mixtures contained 12.5 µL SybrGreen qPCR Master Mix (2×) (Shanghai Ruian BioTechnologies Co., Ltd., Shanghai, China), 0.5 µL each of 10 µM forward and reverse primers (Table S3), 2.5 µL BSA (10 mg/mL), and 8.0 µL sterile, DNA-free water. 1.0 µL standard plasmid or soil DNA extract (1.2–5.1 ng) was added per PCR reaction. The reaction was conducted using an ABI7500 FAST Real-time PCR system. The following program was used for the genes of *nifH*, *chiA*, AOB-*amoA*, AOA-*amoA* and *nirK*: 95°C for 2 min followed by 40 cycles of 95°C for 10 s, 55°C for 20 s and 72°C for 1 min. The following program was used for the genes of *nirS* and *nosZ*: 95°C for 2 min followed by 40 cycles of 95°C for 10 s and 60°C for 1 min. Melting curve and gel electrophoresis analyses were conducted to confirm that the amplified products were the appropriate size. The equation Eff = [10^(−1/slope)^ −1] was used to calculated the amplification efficiencies and resulted in the following values: *nifH* 92%, *chiA* 95%, AOB-*amoA* 87%, AOA-*amoA* 95%, *nirS* 92%, *nirK* 86% and *nosZ* 93%. The gene copy number was calculated using a regression equation that related the cycle threshold (C_t_) value to the known number of copies in the standards. For each soil sample, the qPCR reactions were repeated three times. We added BSA to the PCR reaction mixes to reduce the inhibitory effects of co-extracted polyphenolic compounds in the soil. Additionally, to estimate the possible inhibitory effects of co-extracted polyphenolic compounds, three replicates of PCR for several samples were conducted after adding known amounts of standard plasmid with the soil DNA extract. Inhibitory effects were found to be negligible.

### Data analysis

**Figure 1:**
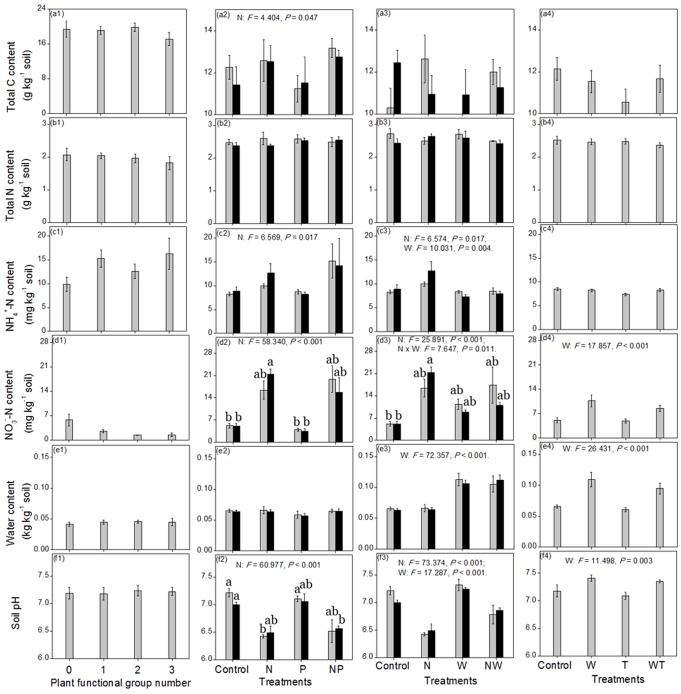
The effects of different treatments on six soil physicochemical indexes. Error bars represent one standard error. For clarity, only the statistical results with *P*<0.05 are shown. 0, 1, 2 and 3 for the x axis in a1–f1 represent the remained PFG number. M, N, P, W and T in a2–f4 represent the treatments of simulated mowing, N addition, P addition, watering, and warming, respectively. In a2–f2 and a3–f3, gray and black columns represent the treatments without and with mowing, respectively. There are 5, 4, 4 and 6 replicates for a1–f1, a2–f2, a3–f3 and a4–f4, respectively.

**Figure 2:**
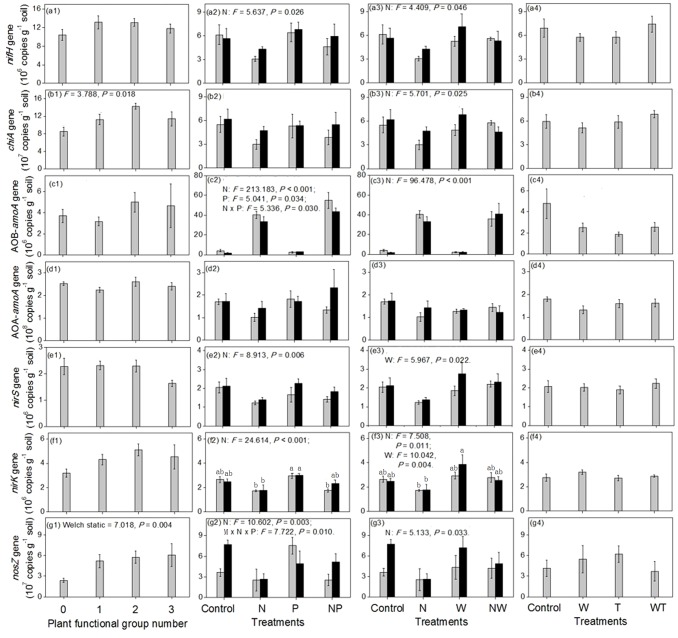
Effects of different treatments on the abundances of seven N-cycling genes. Error bars represent one standard error. For clarity, only the statistical results with *P*<0.05 are shown. 0, 1, 2 and 3 for the x axis in a1–g1 represent the remained PFG number. M, N, P, W and T represent the same treatments as Figure 1. In a2–g2 and a3–g3, gray and black columns represent the treatments without and with mowing, respectively. There are 5, 4, 4 and 6 replicates for a1–g1, a2–g2, a3–g3 and a4–g4, respectively.

To test the influence of PFG number on the soil physicochemical indexes (Fig. 1a1–f1) and the abundance of N-cycling genes (Fig. 2a1–g1) in the first experiment, we used one-way analysis of variance (ANOVA) when the variance among different groups was homogeneous but used the Welch method when the variance was heterogeneous. We also used three-way ANOVA to test the effect of PFG composition on the abundance of each N-cycling gene. For the other three experiments, we used three-way or two-way ANOVA to test the effect of different environmental changes on the soil physicochemical indices (Fig. 1a2–f4) and the abundance of N-cycling genes (Fig. 2a2–g4). For the second and third experiments, post-hoc tests were further made to investigate the difference in soil physicochemical indexes and gene abundances among the eight combinations of experimental treatments. Specifically, the Student-Newman-Keuls and Tamhane methods were used for homogeneous and heterogeneous variances, respectively. Based on these statistical results, we could compare the sensitivities of each N-cycling gene to different environmental changes. Specifically, if the abundance of a given N-cycling gene responded significantly (*P*<0.05) to one kind of environmental change but insignificantly (*P*>0.05) to another kind of environmental change, we would infer that its gene abundance as being more sensitive to the former kind of environmental change than to the latter.

**Figure 3:**
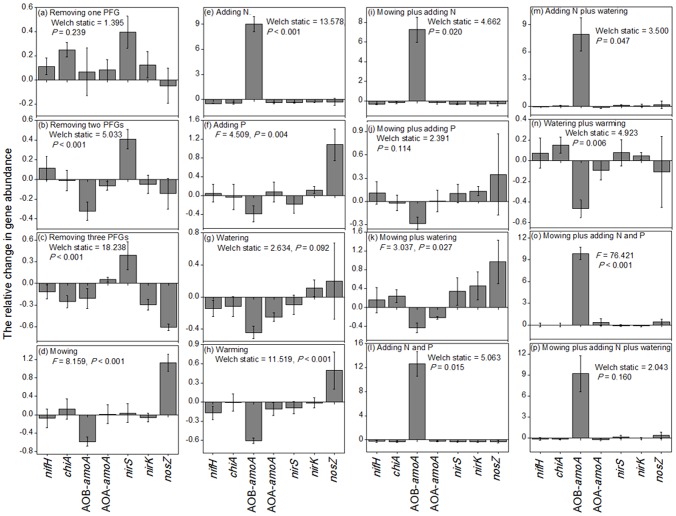
Differential sensitivities of seven N-cycling gene abundances under each of the 16 environmental changes. Error bars represent one standard error.

To compare the sensitivities of different N-cycling gene abundances under each single- or multi-factorial environmental change, for each gene in each treatment plot (excluding the control plot), we calculated its relative change relative to all control plots. Specifically, we first calculated the difference between its abundance and the mean abundance in all control plots and then divided that difference by the mean abundance in all control plots. For the first experiment, we took the treatment with all the three PFGs remaining as the control. After that, for each of the 16 single- or multi-factorial environmental changes (Fig. 3), we used one-way ANOVA to test whether there were significant differences in the relative change in abundance of seven genes.

To compare globally the sensitivities among all environmental changes and all N-cycling genes, for each gene of each of the 16 environmental changes, we first calculated the mean of the relative change in gene abundance (as calculated in the above paragraph) of all replicates, and then calculated the mean absolute of all genes or all environmental changes (Table 1).

**Table 1:** Relative changes in the abundance of various N-cycling genes caused by different treatments.

	*nifH*	*chiA*	AOB-*amoA*	AOA-*amoA*	*nirS*	*nirK*	*nosZ*	Mean absolute
	(fixation)	(mineralization)	(nitrification)	(nitrification)	(denitrification)	(denitrification)	(denitrification)	
oneP	0.111	0.251	0.068	0.084	0.397	0.126	−0.049	0.155
twoP	0.114	−0.012	−0.322	−0.065	0.411	−0.049	−0.145	0.160
threeP	−0.116	−0.254	−0.210	0.053	0.385	−0.298	−0.608	0.275
M	−0.076	0.125	−0.585	0.013	0.034	−0.066	1.127	0.289
N	−0.500	−0.454	9.021	−0.400	−0.399	−0.350	−0.299	1.632
P	0.047	−0.033	−0.389	0.073	−0.188	0.117	1.077	0.275
W	−0.142	−0.118	−0.443	−0.250	−0.096	0.109	0.199	0.194
T	−0.170	−0.007	−0.608	−0.110	−0.088	−0.012	0.496	0.213
MN	−0.299	−0.141	7.255	−0.163	−0.325	−0.328	−0.274	1.255
MP	0.109	−0.020	−0.285	0.006	0.104	0.132	0.347	0.143
MW	0.155	0.241	−0.432	−0.218	0.339	0.458	0.970	0.402
NP	−0.252	−0.292	12.638	−0.217	−0.313	−0.339	−0.295	2.049
NW	−0.090	0.054	7.936	−0.152	0.076	0.054	0.150	1.216
WT	0.072	0.150	−0.465	−0.094	0.077	0.046	−0.107	0.145
MNP	−0.032	−0.003	9.887	0.364	−0.107	−0.121	0.427	1.563
MNW	−0.130	−0.155	9.209	−0.272	0.126	−0.030	0.343	1.466
Mean absolute	0.151	0.144	3.735	0.158	0.217	0.165	0.432	0.714

oneP, twoP and threeP represent removing one, two and three PFGs, respectively. M, N, P, W and T represent the treatments of mowing, N addition, P addition, watering, and warming, respectively.

## Results

### The effect of environmental changes on soil physicochemical indices and gene abundances

In the first experiment, for the six soil physicochemical indices (total C, total N, NH_4_^+^-N, NO_3_^-^-N, water contents and pH), as PFG number decreased from 3 to 0, only NO_3_^-^-N content showed an increasing trend, although its difference among the four PFG gradients was non-significant (*P*>0.05; Fig. 1a1–f1). Three-way ANOVA revealed that the effect of PFG composition on the abundance of each N-cycling gene was non-significant (*P*>0.05). One-way ANOVA showed that only the *chiA* and *nosZ* gene abundances were significantly different among the four PFG gradients (*P*<0.05; Fig. 2b1, g1). Specifically, as PFG number decreased from 3 to 0, *nosZ* gene abundance declined while *chiA* gene abundance first increased and then declined. The abundances of other genes, such as *nifH* and *nirK*, showed similar trends as those for the *chiA* gene.

In the second experiment, for the treatments of mowing, adding N, adding P, and their combinations, only N enrichment had significant effects, increasing soil total C, NH_4_^+^-N and NO_3_^-^-N contents while decreasing soil pH (*P*<0.05; Fig. 1a2–f2). However, while adding N increased soil NH_4_^+^-N content by only about 53%, it increased soil NO_3_^-^-N content by about 317% (independent of mowing and adding P; Fig. 1c2, d2). Meanwhile, only N enrichment significantly decreased the abundances of the genes of *nifH*, *nirS*, *nirK* and *nosZ* (*P*<0.05; Fig. 2a2, e2, f2, g2). While the abundances of the two genes *chiA* and AOA-*amoA* also decreased after N addition, these were non-significant (*P*>0.05; Fig. 2b2, d2). However, N addition significantly increased the abundance of AOB-*amoA* gene and this increase was amplified when P was added concurrently with N (*P*<0.05; Fig. 2c2).

In the fourth experiment, among the treatments of watering, warming, and their combinations, only watering had significant effects, increasing soil NO_3_^-^-N content, water content, and pH (*P*<0.05; Fig. 1a4–f4). These treatments did not significantly alter the abundance of any N-cycling gene (*P*>0.05; Fig. 2a4–g4).

From the third experiment, we detected a significant effect of simultaneous N enrichment and watering on soil physicochemical indexes and gene abundances, beyond the effect of adding N and watering that had already been shown in the second and fourth experiments, respectively. Adding N alone increased soil NH_4_^+^-N concentration significantly relative to the control but simultaneous N addition and watering had only a negligible effect on soil NH_4_^+^-N concentration (Fig. 1c3). Adding N alone considerably increased soil NO_3_^-^-N concentration relative to the control but simultaneous N fertilization and watering increased soil NO_3_^-^-N concentration to a lesser degree (Fig. 1d3). Adding N alone decreased soil pH significantly relative to the control but simultaneous enrichment of N and watering decreased soil pH only slightly (Fig. 1f3). While adding N alone substantially decreased the abundances of *nirS*, *nirK*, and *nosZ* genes relative to the control, adding N together with watering had only a negligible effect on the abundances of these genes (Fig. 2e3, f3, g3).

### The sensitivities of the abundance of each N-cycling gene to different environmental changes

We compared the sensitivities in the abundance of each N-cycling gene to different environmental changes. Among the six manipulation factors (removing PFG, mowing, adding N, adding P, watering and warming), the abundance of *nifH* gene was only significantly changed by adding N (*P*<0.05; Fig. 2a1–4), while the abundances of *chiA* and *nosZ* genes were affected by removing PFG and by adding N (Fig. 2b1–4, g1–4) and AOB-*amoA* gene abundance was affected by adding N alone and by adding P when N was added concurrently (Fig. 2c1–4). Finally, AOA-*amoA* gene abundance was affected by none of these factors (Fig. 2d1–4), while *nirS* and *nirK* gene abundances were affected by adding N and watering (Fig. 2e1–4, f1–4).

We further compared the sensitivities in the abundance of all these genes to different environmental changes. As shown in Table 1, the mean absolute value of the relative change in the seven N-cycling gene abundances in response to adding N alone and the concurrence of adding N with other environmental changes (N, MN, NP, NW, MNP, MNW; see the meaning of these acronyms in the annotation of Table 1) was 1.22–2.05, considerably larger than the mean absolute value for the other ten cases (0.143–0.402).

### The sensitivities of the abundances of different N-cycling genes under each single- or multi-factorial environmental change

To compare the sensitivities of different N-cycling gene abundances to each single- or multi-factorial environmental change, we analyzed whether there were differences in the relative change of these genes abundances. For the three types of environmental changes, including removing one PFG, watering, and simultaneous mowing and adding P (Fig. 3a, g, j), the differences in the relative change in abundance among the seven N-cycling genes were non-significant (*P*>0.05). For the six types of environmental changes, including N addition and the concurrence of N addition with other treatments (Fig. 3e, i, l, m, o, p), the relative change in the abundance of AOB-*amoA* gene was larger than those of the other six genes. For removing two PFGs (Fig. 3b), the absolute values of the relative change in abundance were largest for *nirS* and AOB-*amoA* genes. For removing all the three PFGs (Fig. 3c), the absolute value of the relative change in abundance was largest for *nosZ* gene. For the four types of environmental changes, including mowing, adding P, warming, and simultaneous mowing and watering (Fig. 3d, f, h, k), the absolute values of the relative change in abundance were largest for *nosZ* and AOB-*amoA* genes. For simultaneous watering and warming (Fig. 3n), the absolute value of the relative change in abundance for AOB-*amoA* gene was larger than those for other genes.

We further compared the sensitivities of different N-cycling gene abundances to all of the environmental changes. As shown in Table 1, the mean absolute value of the relative change in abundance under all the 16 environmental changes for AOB-*amoA* gene was 3.73, 0.432 for *nosZ*, and 0.217 for *nirS*, while those for the other four genes were only 0.144–0.165.

## Discussion

Our results indicate that the abundances of different microbes involved in soil N cycling processes respond differentially to various environmental changes of relevance to ongoing global change perturbations. The mechanisms responsible for these idiosyncrasies likely reflect complex feedbacks within the soil – microbe – plant system. In the first experiment, when the PFG number decreased, plants likely absorbed less soil nitrogen, resulting in the observed increase of soil NO3^-^-N content (Fig. 1d1) [30]. In contrast, the content of soil total carbon was so large that it was not significantly altered by the five-year effect of PFG removal (*P*>0.05; Fig. 1a1). However, as PFG number decreased, the amount of labile organic material supplied from plants to soil microbes would likely decrease [49], [50]; this may have contributed to the decrease in the gene abundances of the heterotrophic N-cycling microbes (including *nifH* and *nosZ*; Fig. 2b1, g1). The abundances of the three genes of *nifH*, *chiA* and *nirK* had maximum values in the treatment with two PFGs (Fig. 1), a result that suggests that plants may out-compete soil microbes in some way (e.g., in absorbing trace elements) in the three PFG treatment while plants may provide too little labile organic material to soil microbes in treatments with one and zero PFG. The underlying mechanism for this phenomenon needs to be explored in future research.

Soil pH is a very important ecological factor for soil microbes [51]–[53]. In the second experiment, N enrichment reduced soil pH (Fig. 1f2), an effect that perhaps contributed to the decrease in the abundances of most of the N-cycling genes, including *nifH*, *chiA*, *nirS*, *nirK*, and *nosZ* (Fig. 2a2, b2, e2, f2, g2). Additional factors may have been associated with the negative effect of N addition on the abundances of the N-fixation gene (*nifH*; Fig. 2a2) and the mineralization gene, *chiA* (note that the *P* value was larger than 0.05 in Fig. 2b2 but smaller than 0.05 in Fig. 2b3). These N-fixation and mineralization microbial groups may allocate considerable material/energy resources to increase their ability to acquire N but as a result they were weak in some other abilities, such as acquiring trace elements. Therefore, when soil N was scare, these microbial groups had a competitive advantage. In contrast, when soil N was rich, they were less competitive for these alternative resources [54]–[56].

In contrast to the effects of N enrichment on the abundance of N-fixing genes, adding N increased the abundance of AOB-*amoA* gene (Fig. 2c2), perhaps because it increased the energy source of NH_4_^+^-N for AOB (Fig. 1c2) and because AOB might be resistant to acidified soil [8], [16]. While increased AOB abundance would promote the transformation of NH_4_^+^-N into NO_3_^-^-N, the decreased abundances of denitrification functional groups (*nirS*, *nirK* and *nosZ*) would restrain the transformation of NO_3_^-^-N into NO, N_2_O and N_2_. Meanwhile, precipitation is very low in these steppe ecosystems, so soil NO_3_^-^-N would not be lost through leaching. Therefore, adding N caused soil NO_3_^-^-N content to increase by a larger extent than soil NH_4_^+^-N content (Fig. 1c2, d2). AOB-*amoA* gene abundance was further raised by P enrichment when it coincided with N enrichment (Fig. 2c2). Both N and P are key nutrient resources for all biota and both of them have been found to be limiting elements in these study areas [57]. NH_4_^+^-N (NH_3_) serves both as an energy resource and a basic nutrient element while P is another basic nutrient element needed for biomass construction and energetic metabolism [16]. Thus, the response of AOB to P in the presence of N (but not to P added alone) suggests a scenario in which adding N shifted AOB from being limited by N to being limited by P.

In the third experiment, adding N alone increased soil NH_4_^+^-N content relative to the controls but simultaneous N addition and watering did not (Fig. 1c3). Meanwhile, the AOB-*amoA* gene abundances were almost equal between the two treatments (Fig. 2c3). These results implied that watering promoted the transformation of NH_4_^+^-N into NO_3_^-^-N through stimulation of the activity of AOB-nitrification [58], [59]. This possible mechanism needs to be tested in future studies. Similarly, while adding N alone increased soil NO_3_^-^-N content by a large extent, simultaneous N enrichment and watering increased only modestly (Fig. 1d3). We envision three potential mechanisms for this phenomenon. First, watering may have promoted the leaching of soil NO_3_^-^-N. Second, watering potentially buffered the acidification effect of N addition (Fig. 1f3) and promoted the maintenance of *nirS*, *nirK* and *nosZ* gene abundances (Fig. 2e3, f3, g3), which then promoted the transformation of NO_3_^-^-N into NO, N_2_O and N_2_. Third, the buffered soil pH (Fig. 1f3), the increased soil water content (Fig. 1e3) and the anaerobic environment produced by watering [60] may have concurrently stimulated the activities of these denitrification processes, resulting in lower soil NO_3_^-^-N than when N was added without additional water.

Overall, our results indicate that the abundances of various N-cycling genes responded to distinct and different environmental changes (Fig. 2). Meanwhile, under each single- or multi-factorial environmental change, different N-cycling gene abundances had different sensitivities (Fig. 3), likely reflecting the fact that various N-cycling microbial groups have different physiological traits. For example, different microbial groups depended on carbon or nitrogen sources to a different degree or have different resistance to acidic environments. According to our results, to be effective in detecting or predicting potential changes in any given type of N-cycling process in the context of various global changes, we should pay more attention to the environmental changes that impinge on the particular N cycling gene abundance of interest. For example, we should focus on the loss of plant diversity and N deposition for mineralization processes (Fig. 2b1–b4) but on the inputs of N and P for the nitrification process mediated by AOB (Fig. 2c1–c4). Meanwhile, to be effective in detecting or predicting the shifting trend of the equilibrium state of different N-cycling processes under a certain type of environmental change, we should pay more attention to the process with the most sensitive response in gene abundance for that particular environmental change. For example, our data suggest that we should pay attention to the denitrification process driven by *nosZ* gene as well as the nitrification process mediated by AOB-*amoA* gene under mowing perturbations (Fig. 3d) but pay attention to the nitrification process mediated by AOB-*amoA* gene under simultaneous changes in rainfall and warming (Fig. 3n).

Our integrated results (Table 1) further show that, for all these N-cycling genes, their abundances were most sensitive to N enrichment than to other environmental changes; meanwhile, for all these environmental changes, AOB-*amoA* gene abundance was the most sensitive, followed by two denitrifying gene abundances (*nosZ* and *nirS*) while the other four gene abundances were least sensitive (*nifH*, *chiA*, AOA-*amoA* and *nirK*). Therefore, future attention should be focused especially on N addition and on the bacterial nitrification process. Given that all these functional groups are responsible for the cycling of N, it is not surprising that they were most sensitive to adding N than to other environmental changes. AOB is known to have lower phylogenetic diversity than other N-cycling functional groups [8], [16], helping understand its relatively high sensitivity in gene abundance to various environmental perturbations. This high sensitivity suggests that AOB are a key gatekeeper in the N cycle and implies that AOB might be used as sentinel organisms for environmental changes. Although AOA is another potential functional group driving the ammonia-oxidizing process and they were about 10-fold more abundant than AOB in our steppe study sites, the abundance of AOA-*amoA* gene did not respond significantly to any of these environmental changes (*P*>0.05; Fig. 2d1–d4). This might be because AOA has higher genetic/metabolic diversity and thus stronger resistance to environmental changes than other N-cycling microbial groups. For example, AOA can gain energy from other processes other than ammonia oxidization [61].

In conclusion, by investigating the response of various N-cycling gene abundances to multi-factorial global changes, we successfully compared their sensitivities to various environmental changes and compared the relative responsiveness of different N-cycling gene abundances under given types of environmental changes. However, the extent to which the sensitivities of the *abundances* of different N-cycling genes translate into the sensitivities in *rates* of different N-cycling processes needs to be studied in the future.

## Supporting Information

Figure S1 **The distribution of plots in the second, third and fourth experiments.**
(DOC)

Table S1 **Plant functional groups and their stoichiometric C:N and other properties.**
(DOC)

Table S2 **Design of the first experiment.**
(DOC)

Table S3 **Primer sequences used in real time PCR.**
(DOC)
