# Supplementary material for: Response of the Abundance of Key Soil Microbial Nitrogen-Cycling Genes to Multi-Factorial Global Changes
Source: PLoS One. 2013 Oct 4;8(10):e76500. doi: 10.1371/journal.pone.0076500 (PMC3790715; doi:10.1371/journal.pone.0076500)
Supplement: Figure S1 — The distribution of plots in the second, third and fourth experiments. (DOC) [file pone.0076500.s001.doc]

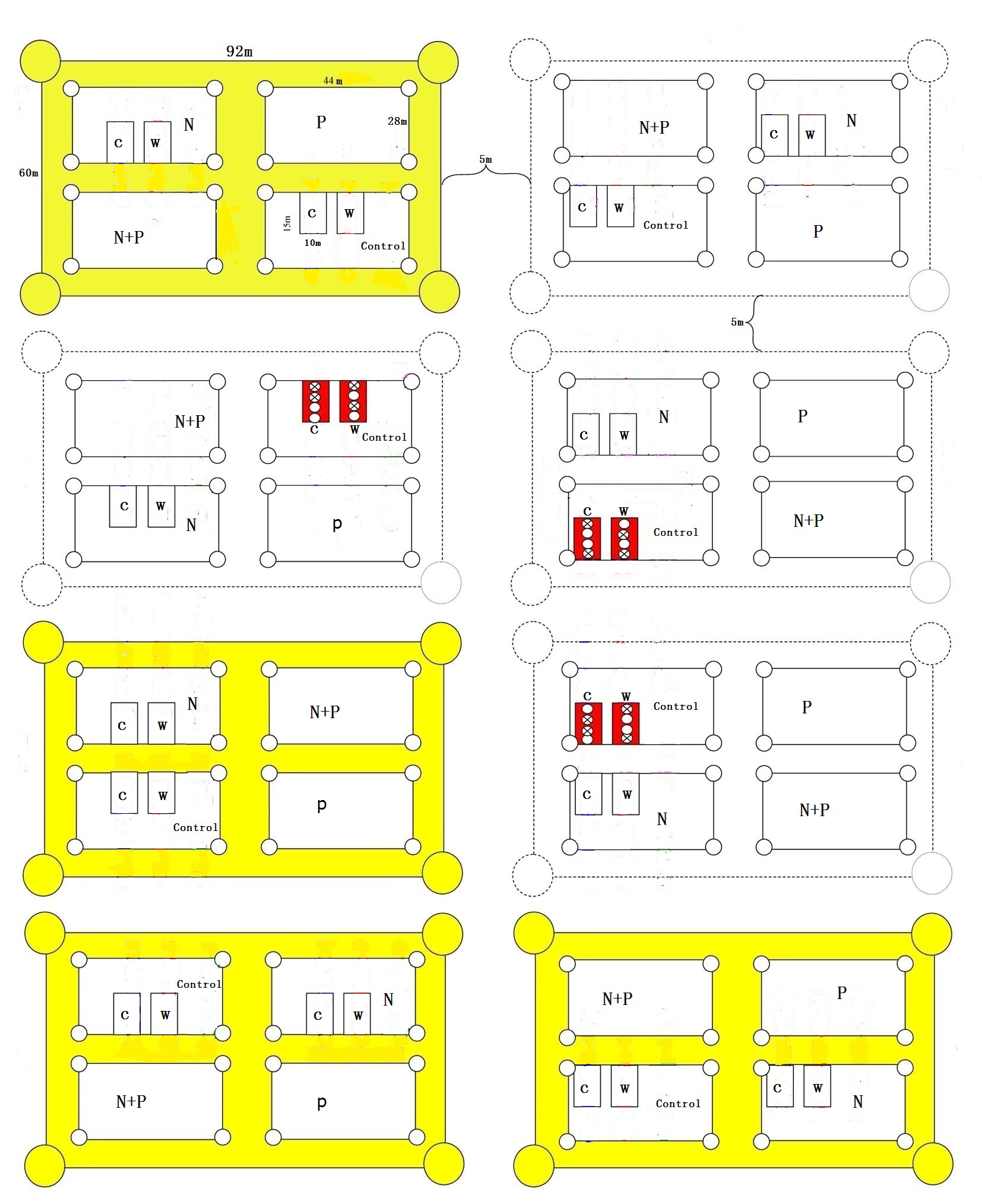


**Figure S1. The distribution of plots in the second, third and fourth experiments.**

The yellow and white primary plots (92m × 60m) represent mowing treatment and control, respectively. The secondary plots (44m × 28m) labeled with control, N, P and N+P represent the treatments of control, N addition, P addition and simultaneous addition of N and P, respectively. The third-level plots (15m × 10m) labeled with C and W represent control (without watering) and watering treatment, respectively. The fourth-level plots were represented with a circle located in the red third-level plots; the circles with a × label and the hollow circles represent warming treatment and control, respectively.
