# Supplementary material for: Response of the Abundance of Key Soil Microbial Nitrogen-Cycling Genes to Multi-Factorial Global Changes
Source: PLoS One. 2013 Oct 4;8(10):e76500. doi: 10.1371/journal.pone.0076500 (PMC3790715; doi:10.1371/journal.pone.0076500)
Supplement: Table S1 — Plant functional groups and their stoichiometric C:N and other properties. (DOC) [file pone.0076500.s002.doc]

**Table S1.** Plant functional groups and their stoichiometric C:N and other properties.

| Plant functional group | No. of sp. per PFG | Representative species | Leaf δ^13^C value (‰)* | C:N (atomic ratio)** | Root/shoot ratio | Aboveground biomass (g.m^-2^) |
| --- | --- | --- | --- | --- | --- | --- |
| Perennial rhizome | 1 | *Leymus chinensis* | -26.13 | 28.99 | 4.0 | 102.10 |
| Perennial bunchgrass | 5-7 | *Stipa grandis;*  *Agropyron cristatum;*  *Cleistogenes squarrosa* | -22.40 | 33.53 | 2.8 | 139.88 |
| Perennial forbs | >20 | *Allium bidentatum;*  *Carex korshinskyi;*  *Potentilla bifurca* | -26.81 | 22.89 | 3.1 | 40.43 |

*Leaf δ^13^C values are related to plant water use efficiency.

**C:N of the aboveground biomass.
