# Supplementary material for: Response of the Abundance of Key Soil Microbial Nitrogen-Cycling Genes to Multi-Factorial Global Changes
Source: PLoS One. 2013 Oct 4;8(10):e76500. doi: 10.1371/journal.pone.0076500 (PMC3790715; doi:10.1371/journal.pone.0076500)
Supplement: Table S2 — Design of the first experiment. (DOC) [file pone.0076500.s003.doc]

**Table S2** Design of the first experiment.

| Treatment no. | PFG gradient | Perennial rhizome | Perennial bunchgrass | Perennial forbs |
| --- | --- | --- | --- | --- |
| 1 | 0 | - | - | - |
| 2 | 1 | + | - | - |
| 3 | 1 | - | + | - |
| 4 | 1 | - | - | + |
| 5 | 2 | + | + | - |
| 6 | 2 | + | - | + |
| 7 | 2 | - | + | + |
| 8 | 3 | + | + | + |

“-” and “+” represented the corresponding PFG being removed and remained, respectively.
