# Supplementary material for: Response of the Abundance of Key Soil Microbial Nitrogen-Cycling Genes to Multi-Factorial Global Changes
Source: PLoS One. 2013 Oct 4;8(10):e76500. doi: 10.1371/journal.pone.0076500 (PMC3790715; doi:10.1371/journal.pone.0076500)
Supplement: Table S3 — Primer sequences used in real time PCR. (DOC) [file pone.0076500.s004.doc]

**Table S3 Primer sequences used in real time PCR.**

| Gene name | Primer name | Primer sequence (5’–3’) | Product size (bp) | source |
| --- | --- | --- | --- | --- |
| *nifH* | *nifH*-F | AAAGGYGGWATCGGYAARTCCACCAC | 458 | Rösch *et al.* 2002 |
|  | *nifH*-R | TTGTTSGCSGCRTACATSGCCATCAT |  |  |
| *chiA* | *chif*2 | GACGGCATCGACATCGATTGG | 409 | Xiao *et al.* 2005 |
|  | *chir* | CSGTCCAGCCGCGSCCRTA |  |  |
| AOB-*amoA* | *amoA*-1F | GGGGTTTCTACTGGTGGT | 491 | Rotthauwe *et al.* 1997 |
|  | *amoA*-2R | CCCCTCKGSAAAGCCTTCTTC |  |  |
| AOA-*amoA* | Arch-*amoAF* | STAATGGTCTGGCTTAGACG | 635 | Francis *et al.* 2005 |
|  | Arch-*amoAR* | GCGGCCATCCATCTGTATGT |  |  |
| *nirS* | cd3AF | GTSAACGTSAAGGARACSGG | 425 | Michotey *et al.*, 2000; Throback *et al.*, 2004 |
|  | R3cd | GASTTCGGRTGSGTCTTGA |  |  |
| *nirK* | *nirK*-1F | GGMATGGTKCCSTGGCA | 515 | Braker *et al.* 1998 |
|  | *nirK*-5R | GCCTCGATCAGRTTRTGG |  |  |
| *nosZ* | nosZ-F | CGY TGT TCM TCG ACA GCC AG | 454 | Throback *et al.*, 2004 |
|  | nosZ-R | CGSACCTTSTTGCCSTYGCG |  |  |
